# Comparative Analysis of Surgery-to-Consultation Ratios Across Surgical Specialties at a Brazilian Tertiary Referral Hospital

**DOI:** 10.7759/cureus.114222

**Published:** 2026-08-09

**Authors:** Amanda Tollini de Moraes, Bella Barreira, Henrique da Costa Marins, Davi Paixão Guimarães, Luiz Fernando Manzoni Lourençone

**Affiliations:** 1 Medical Department, Faculty of Medicine, University of São Paulo, São Paulo, BRA; 2 Medical Department, Bauru Medical School, University of São Paulo, Bauru, BRA; 3 Otorhinolaryngology Department, Bauru Medical School, University of São Paulo, Bauru, BRA

**Keywords:** hospital management, quality improvement, surgical procedures, surgical specialties, tertiary care hospital

## Abstract

Introduction: Evaluating the relationship between outpatient consultations and surgical procedures is essential for hospital management, resource allocation, and healthcare planning. This study aimed to characterize surgical activity at the Hospital for Rehabilitation of Craniofacial Anomalies of the University of São Paulo (HRAC-USP) and the Hospital das Clínicas de Bauru (HC-Bauru), identify the surgical specialties with the highest surgery-to-consultation ratios, and determine the most frequently performed procedures in 2023 and 2024.

Methods: A retrospective, descriptive study was conducted using institutional administrative databases from HRAC-USP and HC-Bauru. Data on outpatient consultations and surgical procedures performed between January 2023 and December 2024 were analyzed. The surgery-to-consultation ratio was defined as the number of surgical procedures divided by the number of outpatient consultations performed by the same specialty within the same calendar year. Because the analysis was based on aggregated institutional data rather than linked patient-level records, this metric represents an institutional activity indicator and should not be interpreted as the proportion of patients progressing from consultation to surgery. Descriptive statistics were used to summarize institutional activity. Ratios were compared between years using the two-proportion Z test, and relative risks (RRs) with 95% confidence intervals (95% CIs) were calculated.

Results: A total of 25,849 outpatient consultations and 3,285 surgical procedures were recorded in 2023, compared with 65,535 consultations and 7,004 procedures in 2024. Although surgical volume increased substantially, the overall surgery-to-consultation ratio decreased from 12.7% to 10.7% (RR = 0.84, 95% CI 0.81-0.87; p < 0.001). Plastic Surgery remained the specialty with the highest surgical volume and showed a significant increase in the surgery-to-consultation ratio, from 41.3% to 86.8% (RR = 2.10, 95% CI 2.02-2.18; p < 0.001). Otorhinolaryngology remained the second-highest surgical specialty, with the ratio increasing from 8.9% to 10.3% (RR = 1.16, 95% CI 1.08-1.24; p < 0.001). Pediatric Surgery ranked third in surgical volume in 2023, whereas Gastroenterology became the third-highest-volume specialty in 2024. The most frequently performed procedures included septorhinoplasty, rhinoplasty, primary palatoplasty, turbinectomy, adenotonsillectomy, otologic microsurgery, and laparoscopic cholecystectomy.

Conclusion: This study provides a comprehensive overview of outpatient and surgical activity within a specialized tertiary referral hospital complex and highlights changes in specialty-specific surgical activity over time. The surgery-to-consultation ratio is a practical institutional indicator of surgical activity relative to outpatient workload and may support hospital planning, resource allocation, and future evaluations of healthcare service organization and surgical productivity.

## Introduction

Surgical care is an essential component of modern healthcare systems and plays a critical role in reducing morbidity, mortality, and long-term disability worldwide. As the demand for surgical services continues to increase, healthcare institutions face the challenge of optimizing care delivery, allocating resources efficiently, and maintaining high standards of quality and patient safety [1]. In this context, the evaluation of surgical activity has become an important component of institutional planning, performance monitoring, and healthcare management.

Assessing the relationship between outpatient consultations and surgical procedures provides valuable information about the operational profile of surgical specialties. The surgery-to-consultation ratio, defined as the number of surgical procedures relative to the number of outpatient consultations within the same specialty, offers a complementary institutional indicator of the balance between outpatient and surgical activity. Although this metric is derived from aggregated administrative data and should not be interpreted as the probability of an individual patient progressing from consultation to surgery, it may help characterize differences in service organization, surgical workload, and resource utilization across specialties [2]. These variations may reflect differences in referral patterns, disease complexity, patient profiles, and specialty-specific clinical practices. Such information may assist hospital administrators in planning operating room capacity, workforce allocation, and healthcare infrastructure.

The importance of monitoring surgical activity has become even more evident following the COVID-19 pandemic. The widespread postponement of elective procedures resulted in a substantial decline in surgical volume worldwide and contributed to the accumulation of patients awaiting treatment [3]. In response, healthcare institutions implemented recovery strategies aimed at restoring surgical capacity and expanding access to surgical care. Evaluating changes in outpatient and surgical activity during the post-pandemic period may therefore provide valuable insights into institutional recovery and evolving patterns of healthcare utilization.

Within the Brazilian Unified Health System (Sistema Único de Saúde - SUS), tertiary referral hospital complexes face the dual challenge of responding to increasing demand for high-complexity surgical care while operating under limited financial and structural resources. This challenge is particularly relevant in institutions that integrate historically specialized services, such as rehabilitation of craniofacial anomalies, with general tertiary hospital services encompassing multiple surgical specialties. In this context, indicators based solely on the absolute number of consultations or procedures may not adequately characterize institutional activity. The surgery-to-consultation ratio provides a complementary measure describing the relationship between outpatient consultations and surgical procedures across specialties and may support institutional planning, resource allocation, and evaluation of healthcare service organization.

Despite the relevance of surgical productivity indicators, relatively few studies have evaluated surgery-to-consultation ratios across multiple surgical specialties in tertiary referral hospitals, particularly within the Brazilian public healthcare system. Comparative analyses of institutional activity indicators may improve understanding of specialty-specific service profiles and contribute to evidence-based decisions regarding healthcare planning, surgical capacity, and resource allocation.

Therefore, the present study aimed to characterize surgical activity at the Hospital for Rehabilitation of Craniofacial Anomalies of the University of São Paulo (HRAC-USP) and the Hospital das Clínicas de Bauru (HC-Bauru) by comparing surgery-to-consultation ratios among surgical specialties during 2023 and 2024. Additionally, the study sought to identify the most frequently performed surgical procedures within each specialty and to describe changes in institutional surgical activity over the study period.

## Materials and methods

This retrospective, descriptive, quantitative study was conducted using administrative data obtained from the HRAC-USP and the HC-Bauru, two tertiary referral hospitals affiliated with the University of São Paulo. Data from the calendar years 2023 and 2024 were analyzed.

Hospital administrative databases were reviewed to identify the number of outpatient consultations and surgical procedures performed by each surgical specialty during the study period. Data were extracted from institutional records maintained by HRAC-USP and HC-Bauru and included all consultations and surgical procedures registered between January 1, 2023, and December 31, 2024.

The variables analyzed included the total number of outpatient consultations, the total number of surgical procedures, surgery-to-consultation ratios, and the most frequently performed surgical procedures within each specialty. For the purposes of this study, the surgery-to-consultation ratio was defined as the number of surgical procedures divided by the number of outpatient consultations performed by the same specialty within the same calendar year, expressed as a percentage. Because analyses were based on aggregated institutional activity data rather than linked patient-level records, this metric represents an institutional activity indicator and should not be interpreted as the proportion of individual patients progressing from consultation to surgery. A single patient could contribute multiple outpatient consultations and/or multiple surgical procedures during the study period.

Descriptive statistics were used to summarize outpatient consultations and surgical procedures by specialty. Results are presented as absolute frequencies and percentages. Surgery-to-consultation ratios were calculated for each specialty and for the institution as a whole. Statistical analyses were performed using R version 4.4.1 (R Foundation for Statistical Computing, Vienna, Austria). Comparisons between proportions were conducted using the two-proportion Z test. Confidence intervals (CIs) for proportions were calculated using the Wald method. Relative risks (RRs), absolute differences between proportions, and corresponding 95% CIs were calculated. Statistical significance was defined as a two-sided p-value < 0.05. Because only aggregated institutional administrative data were available, patient-level independence could not be assessed. Therefore, comparisons between years should be interpreted as comparisons of institutional activity indicators rather than patient-level outcomes.

The study was conducted in accordance with the Declaration of Helsinki and the recommendations of the Brazilian National Health Council. Ethical approval was obtained from the appropriate institutional Research Ethics Committee before the study was conducted.

All data were analyzed in an anonymized and aggregated format. No individually identifiable patient information was accessed or disclosed. Data confidentiality was maintained throughout the study, and access to institutional records was restricted to the investigators involved in the project.

## Results

Between 2023 and 2024, HRAC-USP/HC-Bauru experienced a substantial increase in healthcare activity. The number of outpatient consultations increased from 25,849 to 65,535, while surgical procedures increased from 3,285 to 7,004 (Tables 1-2). Despite the increase in surgical volume, the overall surgery-to-consultation ratio decreased significantly from 12.7% (3,285/25,849; 95% CI, 12.3-13.1) in 2023 to 10.7% (7,004/65,535; 95% CI, 10.5-10.9) in 2024 (absolute difference: -2.0 percentage points; RR = 0.84, 95% CI, 0.81-0.87; p < 0.001), reflecting a proportionally greater expansion of outpatient activity (Table 3).

**Table 1:** Outpatient consultations, surgical procedures, surgery-to-consultation ratios, and most frequently performed surgical procedures by specialty at HRAC-USP/HC-Bauru in 2023 Data are presented as the total number of outpatient consultations and surgical procedures, each specialty’s percentage contribution to institutional activity, surgery-to-consultation ratios (defined as the number of surgical procedures divided by the number of outpatient consultations within the same specialty and calendar year), and the three most frequently performed surgical procedures for each specialty in 2023. HRAC-USP: Hospital for Rehabilitation of Craniofacial Anomalies of the University of São Paulo; HC-Bauru: Hospital das Clínicas de Bauru

Year	Specialty	Outpatient Consultations	% of Total Consultations	Surgical Procedures	% of Total Surgeries	Conversion Rate	Procedure 1 (Quantity/%)	Procedure 2 (Quantity/%)	Procedure 3 (Quantity/%)
2023	Institutional Total	25849	-	3285	-	12.7%	-	-	-
2023	Plastic Surgery	4518	17.5%	1868	56.9%	41.3%	Septorhinoplasty (397/21.2%)	Rhinoplasty (314/16.8%)	Secondary cheiloplasty (270/14.5%)
2023	Otorhinolaryngology	14492	56.6%	1291	39.3%	8.9%	Otologic microsurgery (185/14.3%)	Turbinectomy (157/12.2%)	Adenotonsillectomy (149/11.5%)
2023	Pediatric Surgery	97	0.36%	59	1.8%	60.8%	Gastrostomy (29/49.1%)	Antireflux surgery (19/32.2%)	Unilateral inguinal/femoral hernioplasty (3/5.1%)

**Table 2:** Outpatient consultations, surgical procedures, surgery-to-consultation ratios, and most frequently performed surgical procedures by specialty at HRAC-USP/HC-Bauru in 2024 Data are presented as the total number of outpatient consultations and surgical procedures, each specialty’s percentage contribution to institutional activity, surgery-to-consultation ratios (defined as the number of surgical procedures divided by the number of outpatient consultations within the same specialty and calendar year), and the three most frequently performed surgical procedures for each specialty in 2024. HRAC-USP: Hospital for Rehabilitation of Craniofacial Anomalies of the University of São Paulo; HC-Bauru: Hospital das Clínicas de Bauru

Year	Specialty	Outpatient Consultations	% of Total Consultations	Surgical Procedures	% of Total Surgeries	Conversion Rate	Procedure 1 (Quantity/%)	Procedure 2 (Quantity/%)	Procedure 3 (Quantity/%)
2024	Institutional Total	65535	-	7004	-	10.7%	-	-	-
2024	Plastic Surgery	2712	4.1%	2354	33.6%	86.8%	Septorhinoplasty (400/17.0%)	Rhinoplasty (313/13.3%)	Primary palatoplasty (301/12.8%)
2024	Otorhinolaryngology	15905	24.3%	1641	23.4%	10.3%	Turbinectomy (272/16.6%)	Adenotonsillectomy (219/13.3%)	Otologic microsurgery (209/12.7%)
2024	Gastroenterology	915	1.4%	174	2.5%	19.0%	Laparoscopic cholecystectomy (58/33.3%)	Gastrostomy (32/18.4%)	Umbilical hernioplasty (28/16.1%)

**Table 3:** Statistical comparison of surgery-to-consultation ratios between 2023 and 2024 Surgery-to-consultation ratios were calculated as the number of surgical procedures divided by the number of outpatient consultations within the same specialty and calendar year. The table presents surgery-to-consultation ratios for 2023 and 2024, absolute differences (percentage points), relative risks (RRs), 95% confidence intervals (95% CIs), and p-values for institutional and specialty-specific comparisons.

Comparison	2023 Conversion Rate (95% CI)	2024 Conversion Rate (95% CI)	Absolute Difference	RR (95% CI)	p-value
Institutional Total	12.7% (12.3-13.1)	10.7% (10.5-10.9)	-2.0 pp	0.84 (0.81-0.87)	<0.001
Plastic Surgery	41.3% (39.9-42.8)	86.8% (85.5-88.1)	+45.5 pp	2.10 (2.02-2.18)	<0.001
Otorhinolaryngology	8.9% (8.4-9.4)	10.3% (9.8-10.8)	+1.4 pp	1.16 (1.08-1.24)	<0.001

Plastic Surgery remained the specialty with the highest surgical volume in both years. The number of procedures increased from 1,868 (56.9%) in 2023 to 2,354 (33.6%) in 2024. The surgery-to-consultation ratio increased significantly from 41.3% (1,868/4,518; 95% CI, 39.9-42.8) to 86.8% (2,354/2,712; 95% CI, 85.5-88.1), corresponding to an absolute increase of 45.5 percentage points and a relative increase of approximately 2.1-fold (RR = 2.10, 95% CI, 2.02-2.18; p < 0.001). Septorhinoplasty, rhinoplasty, and cleft-related reconstructive procedures remained among the most frequently performed operations throughout the study period.

Otorhinolaryngology remained the second-largest surgical specialty. The number of procedures increased from 1,291 (39.3%) in 2023 to 1,641 (23.4%) in 2024. The surgery-to-consultation ratio increased significantly from 8.9% (1,291/14,492; 95% CI, 8.4-9.4) to 10.3% (1,641/15,905; 95% CI, 9.8-10.8), representing an absolute increase of 1.4 percentage points and a relative increase of 15.7% (RR = 1.16, 95% CI, 1.08-1.24; p < 0.001). Although otologic microsurgery was the most frequently performed procedure in 2023, turbinectomy became the leading procedure in 2024.

A change in the third-highest surgical specialty was observed between the study years. In 2023, Pediatric Surgery ranked third in surgical volume, accounting for 59 (1.8%) procedures and presenting the highest surgery-to-consultation ratio among all specialties analyzed (60.8%). In contrast, Gastroenterology occupied the third position in 2024, accounting for 174 (2.5%) procedures and demonstrating a surgery-to-consultation ratio of 19.0%. Laparoscopic cholecystectomy was the most frequently performed procedure within this specialty.

Additional surgical activity in 2024 was observed in Thoracic Surgery, Orthopedics, Urology, and Coloproctology, which together contributed to a broader distribution of surgical procedures across specialties. Thoracic Surgery demonstrated a surgery-to-consultation ratio of 18.0%, whereas Urology and Coloproctology presented similar surgery-to-consultation ratios of approximately 10%. Orthopedics showed the lowest surgery-to-consultation ratio among the specialties analyzed (2.2%). These findings highlight substantial variation in surgery-to-consultation ratios across specialties, reflecting differences in clinical profiles and surgical demand.

Overall, the comparison between 2023 and 2024 demonstrated not only a substantial increase in outpatient consultations and surgical procedures but also important changes in the institutional surgical profile, characterized by the continued predominance of Plastic Surgery, a proportional reduction in the contribution of Otorhinolaryngology to the overall surgical volume, and the emergence of Gastroenterology as the third-highest-volume surgical specialty.

## Discussion

Plastic Surgery remained the specialty with the highest surgical volume in both years analyzed. This finding is consistent with HRAC-USP’s role as a national and international referral center for the treatment of cleft lip and palate, craniofacial anomalies, and complex congenital conditions [4]. Procedures such as rhinoplasties, septorhinoplasties, cheiloplasties, and palatoplasties constitute essential components of multidisciplinary treatment protocols and are frequently performed across multiple stages of care [5]. Therefore, the predominance of Plastic Surgery reflects the institutional profile of HRAC-USP as a highly specialized tertiary referral center.

One of the most notable findings was the substantial increase in the Plastic Surgery surgery-to-consultation ratio, from 41.5% in 2023 to 86.8% in 2024. This increase resulted from both a higher number of surgical procedures and a reduction in outpatient consultations. Possible explanations include changes in referral pathways, outpatient organization, preoperative patient selection, expansion of surgical capacity, or prioritization of patients already eligible for surgery. However, these factors were not directly evaluated in the present study and therefore remain speculative. Because this analysis was based on aggregated administrative data, it was not possible to determine which organizational or operational factors contributed to the observed increase.

Otorhinolaryngology remained the second-highest surgical specialty, demonstrating growth in the absolute number of procedures and a modest increase in the surgery-to-consultation ratio. The large number of turbinectomies, tonsillectomies, and otologic surgeries reflects the broad scope of the specialty within HRAC-USP, which is also recognized nationally as a referral center for hearing rehabilitation and cochlear implantation. Likewise, the increase in turbinectomies observed in 2024 may reflect changes in surgical activity or service organization; however, the underlying reasons could not be evaluated using the available administrative data [6].

Another relevant finding was the change in the specialty occupying the third position in surgical volume. While Pediatric Surgery ranked third in 2023, Gastroenterology assumed this position in 2024. This shift may reflect the expansion of HC-Bauru’s participation in surgical care following its incorporation into the hospital complex, together with increased performance of minimally invasive procedures such as laparoscopic cholecystectomy [7]. However, the surgery-to-consultation ratio observed for Pediatric Surgery in 2023 should be interpreted with caution because it was based on a relatively small number of consultations (n = 97) and procedures (n = 59), making this estimate more susceptible to random variation than those obtained from higher-volume specialties.

In addition to the continued predominance of Plastic Surgery and Otorhinolaryngology, increased participation in Thoracic Surgery, Urology, Coloproctology, Orthopedics, and Gastroenterology was observed in 2024. Historically, HRAC-USP has been dedicated primarily to the rehabilitation of craniofacial anomalies, whereas HC-Bauru encompasses a broader range of medical and surgical specialties. The integration of these institutions expanded the diversity of procedures performed within the hospital complex and reflects the transition toward a comprehensive tertiary referral hospital profile. From a hospital management perspective, this diversification may contribute to improved service capacity, integrated patient care, and reduced need for referral to external institutions.

From an administrative perspective, the surgery-to-consultation ratio may contribute to a better understanding of differences in outpatient and surgical activity across specialties and over time. However, because this indicator is derived from aggregated numbers of consultations and surgical procedures rather than linked patient-level trajectories, it should not be interpreted as reflecting patient selection, clinical indications, or the probability that an individual patient progresses from consultation to surgery. Instead, it should be interpreted as an institutional activity indicator that may support hospital planning, monitoring of surgical workload, and resource allocation.

The findings should also be interpreted within the context of the healthcare system's recovery following the COVID-19 pandemic. Several studies have demonstrated that the suspension or reduction of elective procedures during the pandemic resulted in substantial declines in surgical volume and accumulation of patients awaiting treatment [3,8]. Consequently, many institutions implemented recovery strategies aimed at restoring surgical capacity and reducing waiting lists. The simultaneous increase observed in outpatient consultations and surgical procedures between 2023 and 2024 is consistent with this broader recovery process, although the specific contribution of post-pandemic organizational changes could not be evaluated in the present study.

This study has several limitations. First, its retrospective design relied exclusively on institutional administrative databases. Therefore, individual clinical characteristics, disease severity, referral patterns, waiting times, surgical cancellations, postoperative outcomes, and reasons for treatment decisions could not be evaluated. Second, the surgery-to-consultation ratio was derived from aggregated annual counts of consultations and surgical procedures rather than linked patient-level records. Consequently, it should not be interpreted as the probability that an individual patient progresses from consultation to surgery. In addition, consultations and procedures occurring within the same calendar year may involve different patient cohorts because patients evaluated during one year may undergo surgery in a subsequent year, whereas procedures performed during the study period may have resulted from consultations conducted previously. This temporal mismatch is particularly relevant for highly specialized services characterized by long waiting lists and multistage treatment pathways. Therefore, the surgery-to-consultation ratio should be interpreted as an institutional activity indicator describing the relationship between outpatient and surgical workload rather than an individual patient outcome. Furthermore, because repeated consultations or multiple procedures performed in the same patient could not be identified, patient-level independence could not be assessed. Finally, the findings reflect the experience of a highly specialized tertiary referral hospital complex and may not be generalizable to hospitals with different organizational structures or case mixes.

Despite these limitations, the study has important strengths. It provides a comprehensive evaluation of outpatient and surgical activity over two consecutive years in a specialized tertiary referral hospital complex within the Brazilian Unified Health System (SUS). By combining information on outpatient consultations and surgical procedures across specialties, the study offers a practical institutional indicator that complements traditional productivity measures and may support healthcare planning, monitoring of service organization, and resource allocation. Given the limited number of studies evaluating surgery-to-consultation ratios across surgical specialties, these findings contribute to a better understanding of surgical service organization in Brazilian tertiary hospitals and provide a basis for future investigations incorporating patient-level longitudinal data and additional operational indicators [9].

## Conclusions

This study provides a comprehensive overview of outpatient and surgical activity within a specialized tertiary referral hospital complex and highlights the dynamic nature of healthcare service organization across surgical specialties. The observed changes in surgical activity patterns underscore the importance of continuously monitoring institutional performance indicators to better understand healthcare demands and support evidence-based decision-making.

The surgery-to-consultation ratio may serve as a useful institutional indicator for evaluating specialty-specific activity profiles and supporting healthcare planning and resource allocation. Because this metric is derived from aggregated administrative data, it should be interpreted as an indicator of the relationship between outpatient and surgical activity rather than the probability of an individual patient progressing from consultation to surgery. Future studies incorporating patient-level longitudinal data, together with clinical outcomes, waiting times, patient complexity, and resource utilization, are warranted to further improve the understanding of factors influencing surgical activity across specialties.
